# A comparative evaluation of physical properties of CAD/CAM complete denture resins- an in vitro study

**DOI:** 10.1186/s12903-023-03708-2

**Published:** 2024-01-10

**Authors:** Ojasvini Arora, Nabeel Ahmed, Yuliia Siurkel, Vincenzo Ronsivalle, Marco Cicciù, Giuseppe Minervini

**Affiliations:** 1 Department of Prosthodontics and Implantology, Saveetha Dental College And Hospitals, Saveetha Institute Of Medical And Technical Sciences Saveetha University Chennai, Chennai, Tamil Nadu 600077 India; 2 International European University School of Medicine, Akademika Hlushkova Ave, 42В, Kyiv, 03187 Ukraine; 3 Department of Biomedical and Surgical and Biomedical Sciences, Catania University, 95123 Catania, Italy; 4 Saveetha Dental College and Hospitals, Saveetha Institute of Medical and Technical Sciences (SIMATS), Saveetha University, Chennai, Tamil Nadu India; 5 Multidisciplinary Department of Medical-Surgical and Odontostomatological Specialties, University of Campania “Luigi Vanvitelli”, 80121 Naples, Italy; 6 Multidisciplinary Department of Medical-Surgical and Dental Specialties, University of Campania Luigi Vanvitelli, Caserta, 81100 Italy

**Keywords:** CAD-CAM, Complete dentures, Physical properties, PMMA

## Abstract

**Background:**

In dentistry, there is a growing preference for computer-aided design and computer-aided manufacturing (CAD/CAM) systems over traditional laboratory procedures. However, there is not much literature comparing various CAD/CAM materials. Thus, this study aimed to assess and compare the color stability and hardness of gingival and tooth colored milled and 3D-printed acrylic resins.

**Materials and methods:**

Four types of CAD/CAM materials were prepared: 3D-printed pink shade (PP), milled polymenthymethacrylate (PMMA) pink shade (MP), 3D-printed tooth shade (PT) and milled PMMA tooth shade (MT) (*n* = 6). For hardness, disc shaped samples of 15 mm × 2 mm and for color stability, bar shaped samples of 65mmx10mmx2.5 mm were prepared and polished. Vickers hardness test was performed in a microhardness tester. Color stability test was done by immersing in coffee solution and coca cola for 7 days. Day 0 and day 7 measurements were recorded using a digital spectrophotometer and the change in color was calculated. For statistical analysis, one-way ANOVA and Tukey’s post hoc tests were done.

**Results:**

For color stability, milled PMMA was superior to 3D-printed resin samples. Milled pink and tooth shade samples had similar color stability, whereas 3D-printed tooth shade samples were more color stable as compared to pink shade 3D-printed samples. For hardness, milled tooth shade PMMA was the most superior one, followed by 3D-printed tooth shade, whereas pink shade milled PMMA and 3D-printed resin samples had similar hardness values and were inferior to the tooth shade CAD/CAM materials.

**Conclusion:**

Color stability of milled PMMA is superior to that of 3D-printed resins. Hardness of tooth shade milled and 3D-printed resins is more than that of pink shade milled and 3D-printed resins.

## Introduction

The number of edentulous populations is on the rise because of increasing life expectancy throughout the world, especially in developed countries because of better healthcare system and in developing countries because of poor oral health awareness among the general population [1]. Traditionally, complete dentures are fabricated by compression moulding method. However, because of the high polymerization shrinkage and the resultant detrimental effect on the properties of dentures made by compression moulding method, newer techniques and materials were developed, like the injection moulding technique [2]. Even though injection moulding resulted in dentures with much better properties, it was also based on heat polymerization in a water bath under pressure, which involves multiple patient appointments and laboratory steps. Computer-aided design and computer-aided manufacturing (CAD/CAM) for complete dentures was introduced for the first time by Maeda et al. in 1994 [3]. However, it gained popularity only after 20 years when the first clinical study by Goodacre et al. was published in 2012 [4–8]. CAD/CAM has various uses in dentistry like for making crowns and bridges, surgical stents, custom surgical plates, custom implant abutments, occlusal splints, complete dentures, partial dentures, intraoral scanning etc. [9].

It is possible to create CAD/CAM complete dentures in just two appointments [10]. All clinical records are taken at the initial appointment, either in the form of conventional impressions or digital records via intra-oral scanners. The data is moved to the digital dental laboratory, where the full denture is virtually designed [11–13]. The clinician can view and suggest changes in the design if needed before the denture is processed [9]. The dentures can be inserted in the second appointment. This technique may provide elderly patients with considerable advantages, such as lesser visits to the hospital as well as some claims of greater fit and better material characteristics compared to conventionally made dentures [14–16].

CAD/CAM technology allows for the production of dentures in two ways, namely: subtractive and additive methods. The former involves milling the restoration from a prefabricated block of polymethylmethacrylate (PMMA), while the latter entails building up the restoration layer by layer using 3D-printing, stereolithography, or selective laser sintering [17–20]. Using CAD/CAM eliminates the need for reshaping the final product, saving both labor and time. Given the simplicity of digital design, it is preferable to manual techniques [21, 22].

3D-printed complete dentures appear to be a promising treatment option in the coming time [23, 24]. However, it is still considered a newer treatment modality in clinical practice, and not much research has been done comparing 3D-printed and milled complete dentures in terms of their physical properties. The majority of the research has been focussed on assessing only the trueness of fit. Thus, the aim of this study was to assess and compare the hardness and color stability of pink shade and tooth shade milled and 3D-printed acrylic resins. The null hypothesis was that there would be no difference in the hardness and color stability of both the groups.

## Materials and methods

Four groups of materials were tested (Table 1): Group A - Pink 3D-printed acrylic resin (Asiga DentaBASE, Asiga, Sydney, Australia); Group B - Pink milled PMMA (Ivotion Base, Ivoclar Vivadent, Schaan, Liechtenstein); Group C - 3D-printed tooth shade acrylic resin (Asiga DentaTOOTH, Asiga, Sydney, Australia); Group D - Tooth shade milled PMMA (Ivotion Dent, Ivoclar Vivadent, Schaan, Liechtenstein). Twenty-four samples were assessed for microhardness, and the other 24 were evaluated for color stability (*n* = 6).

**Table 1:** Details of the CAD-CAM materials used in the study

Group	CAD/CAM Material	Processing Method	Manufacturer
A	Asiga DentaBASE	3D- printed Pink	Asiga, Sydney, Australia
B	Ivotion Base	Milled Pink	Ivoclar Vivadent, Schaan, Liechtenstein
C	Asiga DentaTOOTH	3D-printed tooth shade	Asiga, Sydney, Australia
D	Ivotion Dent	Milled tooth shade	Ivoclar Vivadent, Schaan, Liechtenstein

### Sample preparation

Standard Tessellation Language (STL) files of a disc with a 15 mm diameter and 2 mm thickness, and a cuboid bar measuring 65 mm in length, 10 mm in width, and 2.5 mm in thickness, were designed in Geomagic software. The disc samples were used for the color stability test, and the cuboid bar samples were used for the Vickers hardness test.

The milled samples were prepared using the PrograMill PM7 milling machine (Ivoclar Vivadent, Schaan, Liechtenstein). After milling was completed, the samples were cut off from the PMMA bank using an acrylic trimming disc bur. The 3D-printed samples were prepared in a 3D printer (Asiga Pro 4 k, Asiga, Sydney, Australia). 3D printing was done with an incremental layer thickness of 100 μm in a horizontal orientation. After the printing process was completed, the samples were thoroughly cleaned by rinsing in 96% ethanol once for 3 minutes and the second time for 2 minutes. After drying the samples, they were post-cured in an ultraviolet light device (Otoflash G171, NK-Optik, Germany) for 10 minutes to complete the curing process.

After all the samples were prepared, they underwent a series of polishing processes. Initially, a tungsten carbide bur was used to finish them, followed by smoothing with a 400-grit silicon carbide abrasive paper for 10 seconds while wet. Finally, the samples were fine-polished for 30 seconds with the help of a slurry of pumice and water on a lathe machine. Finally, all the samples were cleaned with distilled water in an ultrasonic cleaner for 10 minutes, thoroughly rinsed, and air-dried. Sample preparation and polishing were done by the same operator.

### Color stability test

**Fig. 1:**
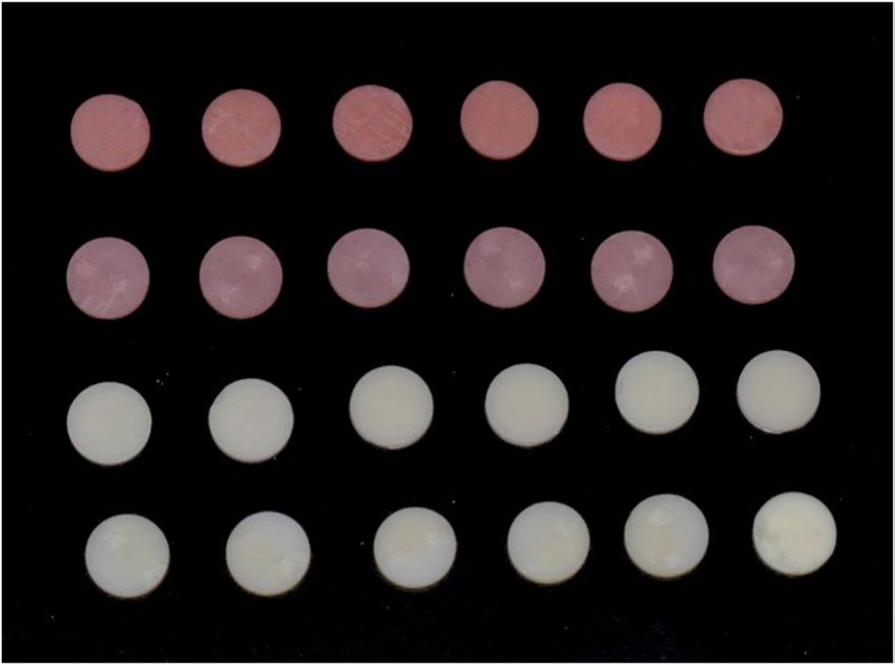
Samples used for color stability test

Disc shaped samples of dimensions15 mm × 2 mm were used (Fig. 1). In order to determine the Commission International de I’Eclairage (CIE) colour parameters L*, a*, and b* for each specimen, a digital spectrophotometer (CM-5 Konica Minolta, Tokyo, Japan) was used. Color measurements for all the samples were recorded before immersing in coloring media on Day 0 three times, and the means were noted as L0*, a0*, and b0*. After that, the specimens were submerged in the staining solutions. One was a coffee solution (8 g of coffee from Nescafe Classic, Nestle USA, dissolved in boiled 0.5 L of distilled water), and the other was Coca Cola (The Coca Cola Company, USA). The solutions were kept at room temperature in a dark environment for seven days, and throughout the test, the staining solution was replaced every day. After 7 days of immersion, the samples were washed with water for a duration of five minutes, left to dry in the air, and then the measurements of their color were documented.

Data were noted as L1*, a1*, b1*. The formula ΔE (ΔE*_ab_ = ΔE_76_) was employed to determine the alterations in the color of the specimens: ΔE*_ab_ = √(ΔL)^2^ + (Δa)^2^ + (Δb)^2^.

In order to evaluate the changes in the color of dental resins, the CIE L*a*b* color space is frequently utilized, with the 1976 CIE being a recommended reference according to the ISO/TR 28642:2016 standard. The degree of color change is represented by ΔE (ΔE_ab_ or ΔE_76_). Two thresholds have been established to specify the extent of color change: the perceptibility threshold (PT), which is the minimum amount of color change that can be detected by 50% of observers, and the acceptability threshold (AT), which is the maximum amount of color change that 50% of observers can accept under controlled conditions.

### Hardness test

**Fig. 2:**
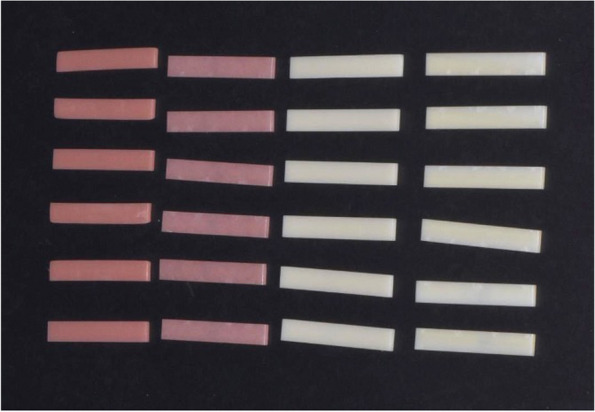
Samples used for harness test

Bar-shaped samples of dimensions 65 mm × 10 mm × 2.5 mm were used (Fig. 2). The surface hardness of the specimens was assessed immediately after they were removed from the distilled water, using the Vickers hardness number (VHN). To perform this test, a Micro Hardness Tester (Shimadzu HMV-G, Kyoto, Japan) was utilized, which employed a square-based pyramid indenter with a 300 g load and a 15-second dwell time. In order to ensure precision, three notches were created on every sample. The resulting pyramids were examined, and their diagonals were measured to determine the VHN. The final VHN was an average of the three individual VHNs.

For statistical analysis, one-way ANOVA test and Tukey’s post hoc analysis was done (*p* < .05). All statistical tests were performed using SPSS version 23.0 (IBM, USA).

## Results

**Fig. 3:**
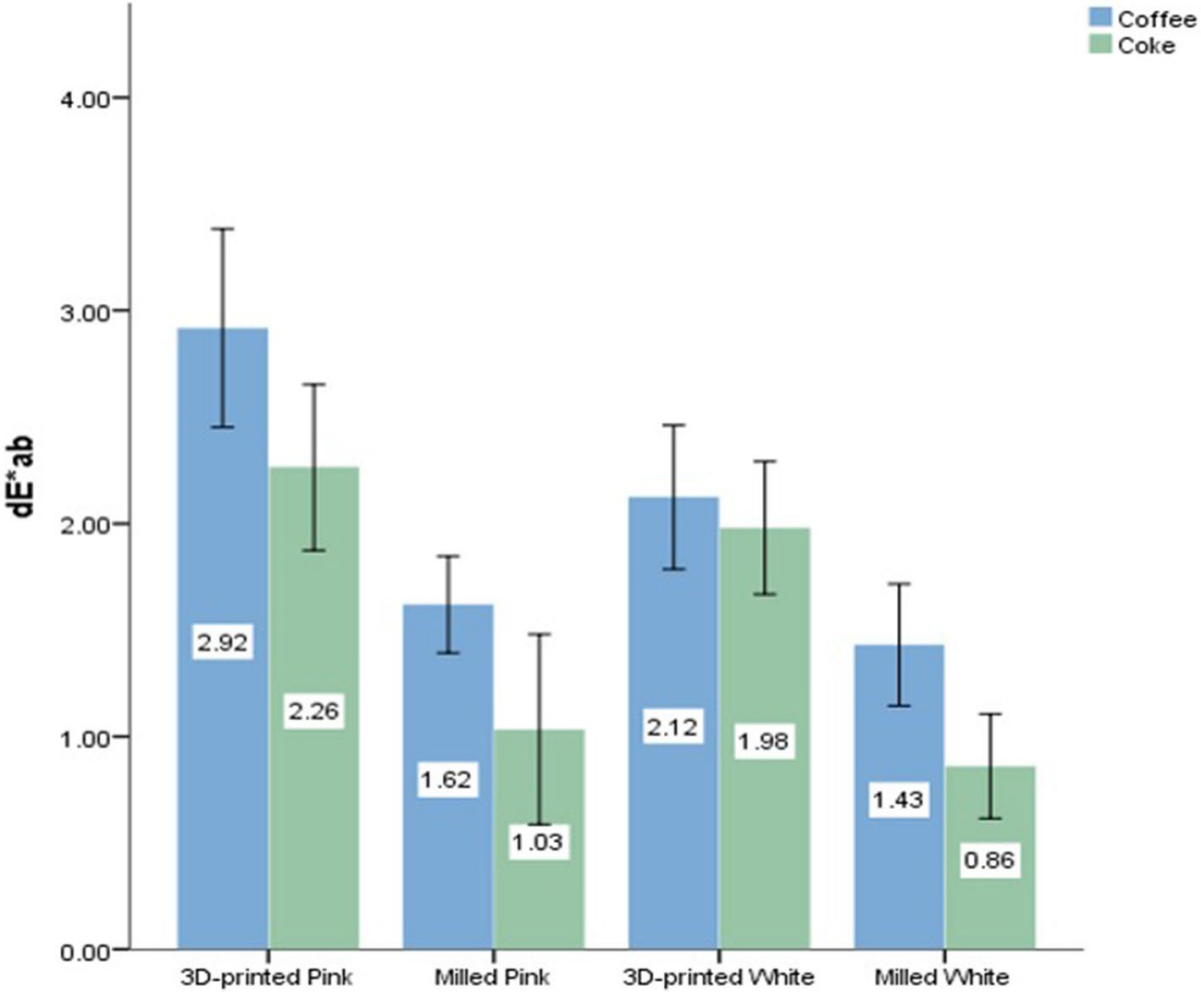
Bar graph showing mean ΔE*ab values in coffee solution and coca cola for the CAD-CAM materials used in the study

**Fig. 4:**
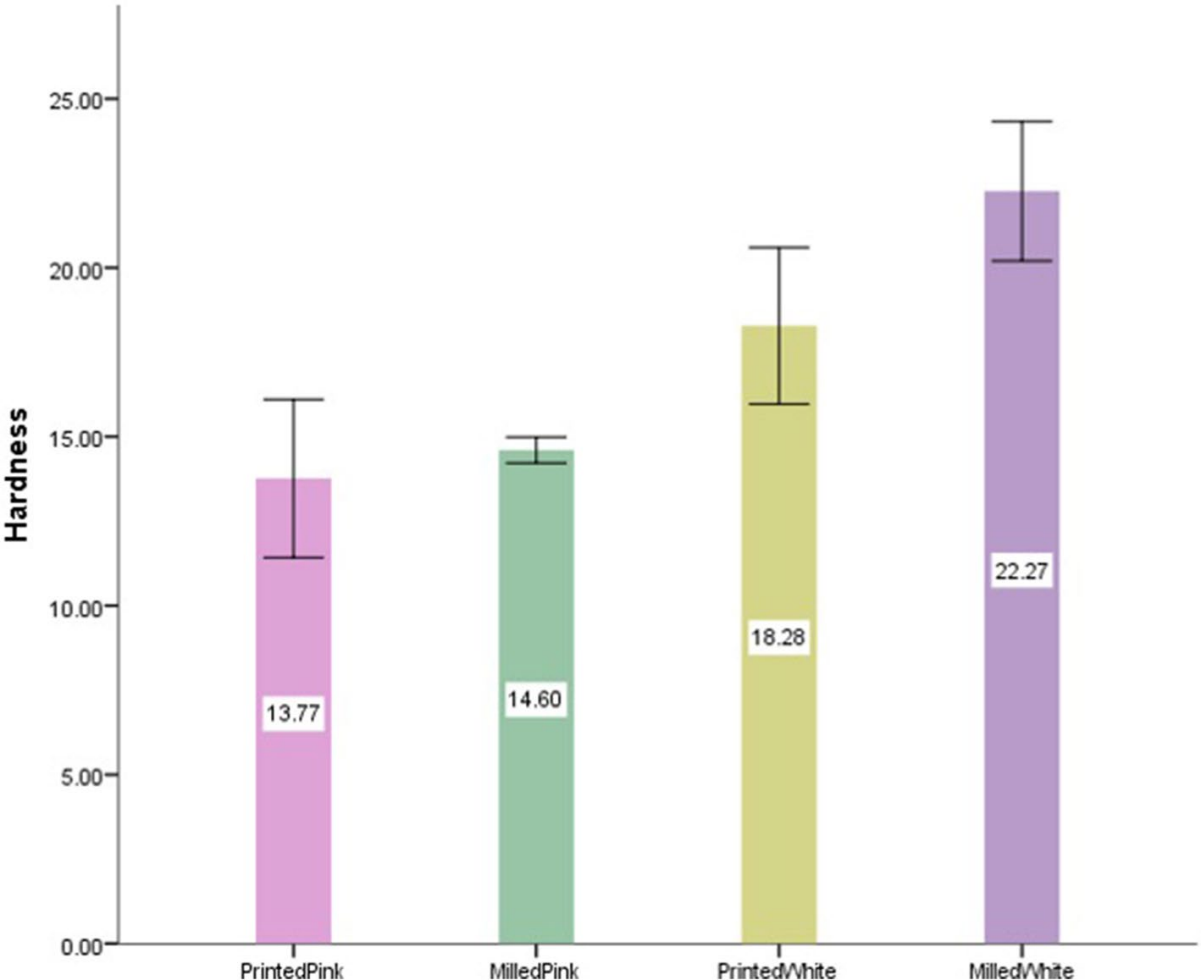
Bar graph showing mean VHN values for the CAD/CAM materials used in the study

The results obtained are shown in Tables 2 and 3 and Figs. 3 and 4.

**Table 2:** Mean values for hardness (in VHN) and mean (ΔE*ab) values in coffee solution and coke for the various CAD-CAM materials used in the study

CAD/CAM Material	Hardness (VHN)	Color Satbility (ΔE*ab)	
		Coffee	Coke
3D-printed Pink	13.76 ± 2.22	2.91 ± 0.18	2.26 ± 0.15
Milled Pink	14.6 ± 0.36	1.62 ± 0.09	1.03 ± 0.18
3D-printed White	18.28 ± 2.21	2.12 ± 0.13	1.98 ± 0.12
Milled White	22.26 ± 1.96	1.43 ± 0.11	0.86 ± 0.09
Significance (one-way ANOVA)	*p* < 0.05	*p* < 0.05	*p* < 0.05

**Table 3:** Tukey’s post hoc analysis values (*p*-value) for the various subgroups. PP- Printed Pink, MP- Milled Pink, PT- Printed Tooth, MT- Milled Tooth

CAD/CAM Material	Hardness	Color Stability	
		Coffee	Coke
PP vs MP	.864	.000	.000
PP vs MT	.002	.000	.150
PP vs PT	.000	.000	.000
MP vs PT	.013	.009	.000
MP vs MT	.000	.385	.490
PT vs MT	.007	.001	.000

As for color stability, milled PMMA was superior to 3D-printed resin samples. Milled pink and tooth shade samples had similar color stability, whereas 3D-printed tooth shade samples were more color stable as compared to pink shade 3D-printed samples.

For hardness, milled tooth shade PMMA was the most superior, followed by 3D-printed tooth shade, whereas pink shade milled PMMA and 3D-printed resin samples had similar hardness values and were inferior to the tooth shade CAD/CAM materials.

## Discussion

The study was conducted to assess and compare the color stability and hardness of different CAD/CAM materials used for the fabrication of complete dentures. The results showed that milled PMMA was generally superior to 3D-printed PMMA. However, in terms of hardness, milled pink shade and 3D-printed pink shade were similar, and in terms of color stability, milled pink shade and tooth shade were similar. Thus, the null hypothesis that there would be no significant difference in the physical properties of these materials was partially rejected.

Color stability of a material can decrease if there is increased surface deterioration. A possible explanation for the surface deterioration is the filler content of the resin. A lower filler content leads to higher surface deterioration [25]. 3D-printing resins typically contain fewer inorganic filler particles than other types of resins, as this helps to maintain low resin viscosity during the printing process and achieve a smooth surface finish [26, 27]. However, this lower filler content can also make the resin less wear-resistant and more susceptible to surface deterioration over time [28]. Additionally, the settling down of filler particles when the resin is stored can lead to non-homogeneous layers during printing, leading to impaired polymerization and further exacerbating surface deterioration [29]. Surface deterioration is also linked to the residual monomer content of the resin. Denture resins with high levels of residual monomer may experience water sorption and expansion, leading to degradation of the surface and mechanical properties [30]. Absorption of water can also help pigments to stick to the resins or move around within them. Berli et al. reported higher water sorption for 3D-printed resins in their study [31]. It is also possible that the 3D-printed resins used in the study had higher residual monomer content, which could have contributed to their surface deterioration and absorption of color pigments with water. However, there is currently no concrete research to support the idea that 3D-printed resins have higher levels of residual monomer content than other types of resins. Therefore, it is important to approach this theory with caution until further research can be done to confirm or refute this speculation. All of these factors may have contributed to the decreased color stability observed in the 3D-printed resin samples [32].

The term “hardness” refers to how much a material can resist being locally deformed through mechanical means such as abrasion or indentation [32–36]. If dentures are made from a material with low surface hardness, they are more likely to sustain damage from mechanical brushing, which can lead to plaque build-up and discoloration, ultimately reducing their lifespan [37]. In the current study, milled tooth shade had highest hardness, followed by 3D-printed tooth shade, and the least and similar hardness was of pink shade milled and 3D-printed resins. Srinivasan M et al. found no significant difference in the hardness of milled and 3D-printed resins [38]. However, the milled samples used in their study were of a different brand. In the study by Prpic et al., even though they observed that 3D-printed resins had lower hardness values, an interesting point was highlighted that the different brands of milled and printed materials showed different properties irrespective of their polymerization process, and it has to do with the density of the material [39].

## Limitations and future scope

Simulation of oral conditions and aging of the samples was not done in the current study. Different brands of CAD/CAM materials should be assessed in future studies. The impact of different orientations used during 3D printing should also be evaluated in future studies. Furthermore, the results of this study should be confirmed by clinical trials.

## Conclusion

1. Color stability of milled PMMA is superior to that of 3D-printed resins.
2. Hardness of tooth shade milled and 3D-printed resins is more than that of pink shade milled and 3D-printed resins.
3. Tooth shade 3D-printed resin is superior to pink-shade 3D-printed resin in terms of color stability and hardness.

## Data Availability

The data will be available on reasonable request from the corresponding author.
